# Development of SpyTag/SpyCatcher-Bacmid Expression Vector System (SpyBEVS) for Protein Bioconjugations Inside of Silkworms

**DOI:** 10.3390/ijms20174228

**Published:** 2019-08-29

**Authors:** Jian Xu, Tatsuya Kato, Enoch Y. Park

**Affiliations:** 1 Laboratory of Biotechnology, Research Institute of Green Science and Technology, Shizuoka University, 836 Ohya, Suruga-ku, Shizuoka 422-8529, Japan; 2 Laboratory of Biotechnology, Graduate School of Integrated Science and Technology, Shizuoka University, 836 Ohya, Suruga-ku, Shizuoka 422-8529, Japan; 3 Laboratory of Biotechnology, Graduate School of Science and Technology, Shizuoka University, 836 Ohya, Suruga-ku, Shizuoka 422-8529, Japan

**Keywords:** protein conjugation, protein expression, SpyTag/SpyCatcher, bacmid, silkworm

## Abstract

Protein conjugations at post-translational levels are known to be essential to protein stability and function. Recently, it has been proven that the split protein CnaB2 (SpyTag/SpyCatcher, ST/SC) from *Streptococcus pyogenes* can induce covalent conjugation rapidly and efficiently under various conditions. The protein of interest fused with the split protein SC/ST could be assembled spontaneously. In light of this finding, we introduced the ST/SC protein coupling concept into the silkworm-bacmid protein expression system (SpyBEVS). As a proof of concept, we first examined and confirmed that a competent ligation occurred between ST/SC-fused protein partners in vitro in cultured silkworm cells and in vivo in silkworm larvae by co-infection of several recombinant baculoviruses. The protein conjugation could be also achieved sufficiently by a simple one-step mixture of purified ST/SC-tagged peptide-protein pairs in vitro. Given the flexibility and robustness of silkworm-BEVS, our results on SpyBEVS show an alternative method for enabling the production of protein decorations in vitro and inside of silkworms.

## 1. Introduction

The production of protein complexes with desired functions has always been a challenge by using prokaryotic (e.g., *Escherichia coli*) or even advanced eukaryotic protein expression systems using yeasts, cultured insect cells, or mammalian cells as hosts [1,2,3]. One key merit of the bacmid expression vector system (BEVS) is its capability of expressing multiple proteins of interest (POIs) simultaneously via co-infection/co-transfection of several recombinant bacmids together [4]. Recently, BEVS using lepidopteran insects, e.g., silkworm (*Bombyx mori*), has enabled us to produce proteins of interest to a higher level with both a satisfactory quantity and quality, where the resulting POIs hold most desirable posttranslational modifications, especially glycosylation, which is considered to be essential for glycoproteins [5,6,7,8,9]. Those advantages should contribute greatly to reducing the cost of the commercial proteins, such as enzymes and vaccines in the market, although several issues such as the development of purification methods and the safety risk (e.g., contaminations form either insect- or baculovirus-derived substances) remain to be clarified. Further, several interesting technologies such as RNA interference (RNAi) and gene knock-out techniques have been incorporated to endow BEVS with greater flexibility in cultured lepidopteran cells and mammalian cell lines [10,11,12]. Those approaches (RNAi-BEVS, baculovirus-inducible CRISPR-Cas9) have made it possible to trigger modifications or decorations posttranslationally as needed within cells relying on the modification of the responsible enzymes [10,11,12].

The protein-protein interactions, for instance, the affinity binding (noncovalent) between streptavidin and biotin is considered as the strongest non-covalent interaction which has been utilized as a promising protein for engineering performance by genetic fusion approaches and has already been commercialized as a platform for protein purifications [13]. The limitation of the streptavidin–biotin system is that the interaction is molecular motor- and force-sensitive, and could be broken in various harsh conditions [14]. On the other hand, covalent bonds mechanically formed between specific interns or split-proteins are considered steadier and more useful in rationally engineering artificial proteins with complex topologies [15]. The unique isopeptide bond was formed spontaneously between proximal lysine (Lys) and asparagine (Asn) or aspartic acid (Asp) residue in some bacterial proteins [16]. Recently, a novel (second generation) covalent isopeptide bond formation has been recovered in Lys31 and Asp117 residue from two split domains of *Streptococcus pyogenes* fibronectin-binding protein FbaB (SpyFbaB), referred to as SpyTag/SpyCatcher [17]. The engineered peptide partners, so-called “molecular superglue” have rapid and stable amino coupling features and can tolerate various even harsh conditions of salt concentration, temperature, and pH [17]. Until now, several efforts have been carried out to counter the relatively slow reaction for the development of SpyTag/SpyCatcher chemistry-based applications as a novel tagging strategy, focusing on the screening of the variants of peptides [SpyTag002 (13-residue peptide)/SpyCatcher002 (113-residue peptide)] to form constant and rapid reaction and the establishment of an optimized platform for the protein display, labeling, and purification [18,19,20,21,22,23]. In the past decade, a growing body of literature has conclusively shown the critical applications of SpyTag/SpyCatcher largely based on bacteria, yeasts, and cultured mammalian cells [24,25,26,27,28,29,30]. However, it is still elusive if the SpyTag/SpyCatcher is compatible with insects or its derived cultured cells. Herein, in the light of those findings, we have introduced the improved SpyTag002/SpyCatcher002, thereafter ST/SC [18] bioreactive conjugation concept into the silkworm-bacmid protein expression system (SpyBEVS) in order to investigate the protein posttranslational modification inside of silkworms. From our results, we confirmed that protein conjugation could be achieved sufficiently in vivo and in vitro by co-infection methods and a simple one-step mixture of purified diverse SpyTag/SpyCatcher-tagged protein partners, respectively. To the best of our knowledge, our study is the first effort to combine these two systems using cultured silkworm cells and silkworm individuals, either larvae or pupae, as a biofactory for spontaneous protein assembly. The established SpyBEVS in the current work should share a useful well-designed scheme for the application of in vitro and in vivo protein engineering.

## 2. Results

### 2.1. Establishment of ST/SC-Bacmid Expression Vector System (SpyBEVS)

**Figure 1:**
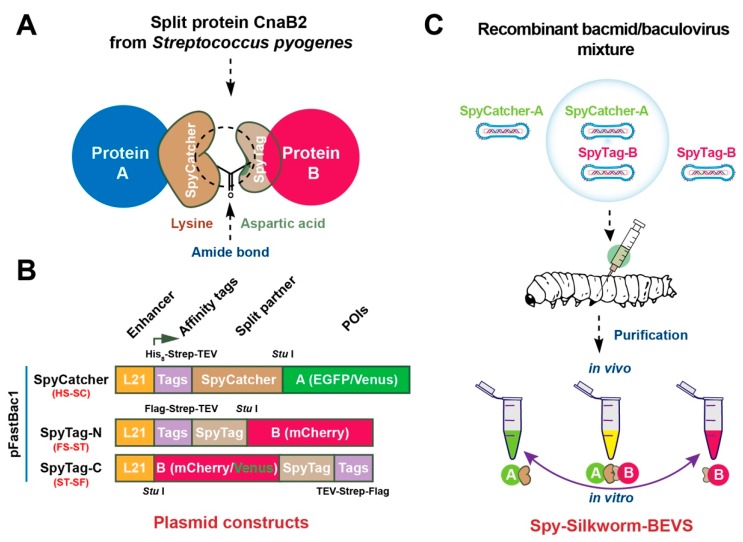
Schematic design of SpyTag/SpyCatcher-Bacmid/Baculovirus expression vector system (SpyBEVS). (**A**) Lysin-Aspartic acid amide bond formation between SpyTag/SpyCatcher-fused (ST/SC) partners. (**B**) Expression (pFastBac1) constructions of SpyCatcher and SpyTag (N/C-terminus). L21, a 5′ untranslated leader sequence (21 bp) from a lobster tropomyosin cDNA as an enhancer to improve the protein expression level in BEVS [31]; His_8_, 8 × histidine tag; Strep, Strep-tag; TEV, a recognition site of TEV protease; FLAG, DYKDDDDK-tag. GGGS linker is presented between each tag to increase the plasticity of whole proteins. As shown, enhanced green fluorescent protein (EGFP), Venus, or mCherry proteins were employed as model proteins for each construct to be investigated in the ligation assay. (**C**) The experimental flow of Spy-Silkworm-BEVS using silkworm instar larvae. Recombinant baculoviruses containing ST/SC-fused proteins of interest were generated by Bac-to-Bac system. The silkworm 5th instar larvae were then injected with single or mixed recombinant bacmids or recombinant baculoviruses to produce ST/SC-fused proteins for in vitro (after protein purification) and in vivo (co-expression in silkworm via co-infection methods) ligation, respectively.

As demonstrated in Figure 1A, the isopeptide bond formed covalently between Lys and Asp residues to form SpyTag: SpyCatcher complex. To investigate the possibility whether the genetic ST/SC-fused protein partners could be sufficiently expressed and coupled in BEVS, we first performed the rational design of several constructions to be used for generating the recombinant baculoviruses. In this study, we intend to express reporter POIs fused with N-terminal SC or N/C terminal ST in pFastBac vector as shown in Figure 1B. To facilitate the expression and purification of POIs, 8 × histidine (His8), Strep-tag II and FLAG tags were added to the ST/SC expression cassette designated as His8-Strep-tag II (HS)-SC and Flag-Strep-tag II (FS)-ST/ST-SF (Strep tagII-Flag), respectively. The genes encoding reporter proteins, such as green fluorescent protein (EGFP), Venus and mCherry proteins, were inserted and genetically fused with ST/SC, respectively. The recombinant *B. mori* baculoviruses (rBmNPVs) were then generated and examined either in cultured silkworm Bm5 cells or silkworm larva or pupa individuals. Single infection and co-infection mixture methods were employed to assess the uncoupled and conjugated ST/SC POI products in vitro and in vivo. The schematic diagram and experimental flow are depicted in Figure 1C.

**Figure 2:**
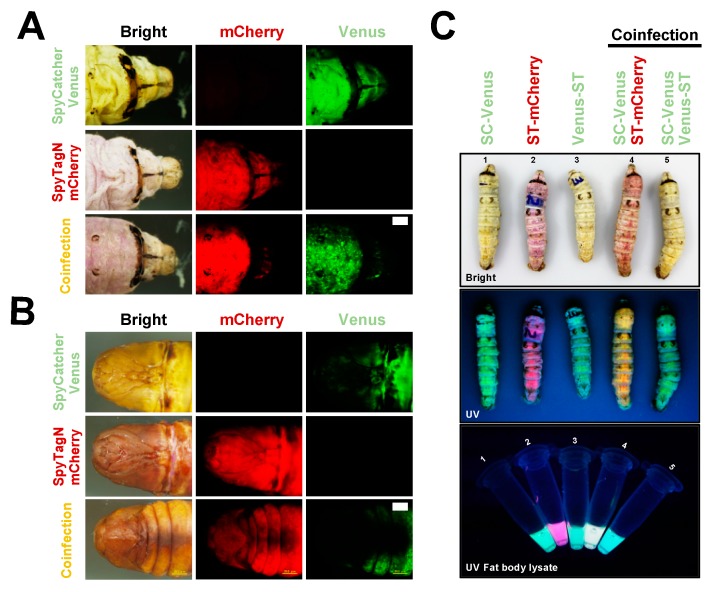
Production of ST- and SC-tagged proteins in silkworm-BEVS. Recombinant bacmid (s) from ST/SC constructs, SC-Venus and ST-N-mCherry were pre-mixed with DMRIE-C transfection reagents and then injected into the third day of silkworm 5th instar larvae (**A**) and silkworm pupae (**B**). The fluorescent signals of EGFP, Venus or mCherry were verified under ultraviolet light (UV). Meanwhile, the whole silkworm larvae and lysates of fat body tissue at 5 d post-infection (dpi) of recombinant baculoviruses were also investigated for fluorescent signals (**C**: upper panel, larvae body under bright light; middle panel, larvae body under UV light; fat body lysates under UV light). All the corresponding expressions could be verified based on the colors of the epidermis or the crude lysate of fat body tissue.

Subsequently, the expression level for SC/ST constructs was investigated in silkworm individuals. The silkworm-bacmid expression system was then employed to investigate the abundance of protein expressed in vivo. Recombinant BmNPV bacmids from ST/SC constructions, SC-Venus ST-mCherry, and Venus-ST were mixed (as indicated in Figure 2) with transfection reagents and injected into the third day of silkworm instar larvae (Figure 2A) and silkworm pupae (Figure 2B). Six days post-infection (dpi), the fluorescent signals from expressed Venus or mCherry proteins were verified under the UV light. Further, the whole silkworm larvae and the lysates from baculovirus-(co)infected fat body tissue at five dpi of indicated rBmNPVs were also investigated for fluorescent signals (Figure 2C). As demonstrated, we validated the expression of all designed ST/SC constructs based on the fluorescent signals. When co-injected with recombinant viruses expressing SC-Venus and ST-mCherry, it is obvious that both green and red fluorescent signals were detected in both silkworm larva and pupa models under UV light. Moreover, we can easily understand those expressions judging from the colors of the epidermis or the crude lysate of fat body tissue, green or pink, at five or six dpi. The results obtained here indicate the success and usability of designed constructs for silkworm-BEVS.

### 2.2. SpyBEVS Enables In Vitro and In Vivo Ligation of ST/SC Protein Pair

**Figure 3:**
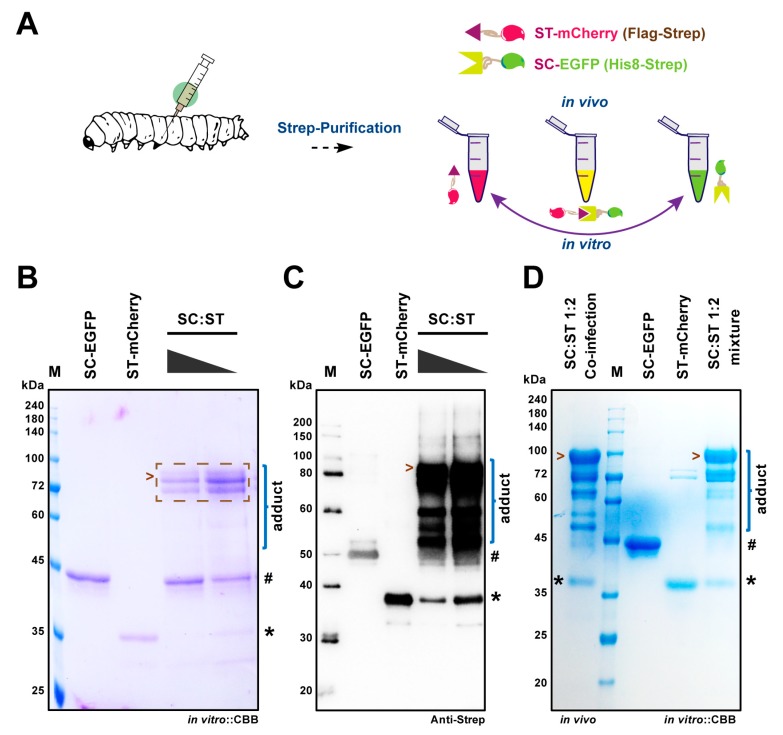
In vitro and in vivo protein ligation of ST/SC partners in silkworm-Spy-BEVS. (**A**) Diagram of expression and ligation of ST-mCherry and SC-EGFP proteins in silkworm-BEVS. Each protein was purified from the fat body of infected fat body and mixed in a decreased ratio of SC-EGFP/ST-mCherry (SC/ST) to investigate the in vitro ligation activity as demonstrated in (**B**) (CBB staining) and (**C**) (Western blot analysis by anti-Strep tag). The in vivo ligation was tested by co-infecting silkworm larvae with two recombinant baculoviruses of SC-EGFP and ST-mCherry (SC/ST = 1:2) together. The resulting products were purified by Strep-column and assessed by SDS-PAGE (**D**). # and * indicate SC-EGFP and ST-mCherry, respectively. Ligated products were marker by the brace and the completed adduct was marked by arrows.

To investigate the resulting ST/SC protein pair, we then assayed the efficiency of conjugation in vitro using the purified SC-EGFP and ST-mCherry proteins or in vivo via directly verifying the protein products from the co-infected silkworm larvae as shown in Figure 3A. In this study, we rationally designed His_8_-Strep-tag II (HS)-tagged SpyCatcher and Flag-Strep-tag II (FS)-tagged SpyTag so that we can extract the ST/SC-labelled proteins by specific tag affinity purification processes. When SC-EGFP and ST-mCherry mixed together in PBS at room temperature for 1 h, an adduct was shown after separation by both SDS-PAGE (Figure 3B) and Western blot (Figure 3C) with decent specificity, suggesting the successful conjugation between the ST/SC protein pair. It also exhibited an increased amount of adduct when baring more ST-mCherry, as indicated in Figure 3B,C. To prove the in vivo bioconjugation, the lysates of fat body tissues were subjected to Strep-purification, by which technically all products holding Strep-tag should be co-pulled down into the elution fraction. As expected, the SDS-PAGE results from Figure 3B,D verified that same patterns regarding the in vitro ligation products were gained inside of silkworm. Moreover, the ratio of viruses used for co-infection between SC-EGFP and ST-mCherry (1:2) met well with the ratio of proteins expressed. It has been also noted that the overexpressed ST-mCherry proteins saturated the relatively lower expressed SC-EGFP in the silkworm to make a sufficient assembled product, SC-EGFP:ST-mCherry. An interesting observation as displayed in this figure is that, several intermediate/incomplete products also presented in both in vivo and in vitro models, which was probably caused by the protein degradation or alternations in conformation after ligation of SC-EGFP and ST-mCherry.

### 2.3. Application of SpyBEVS as a Post-Translational Protein Conjugation and Labeling Platform

**Figure 4:**
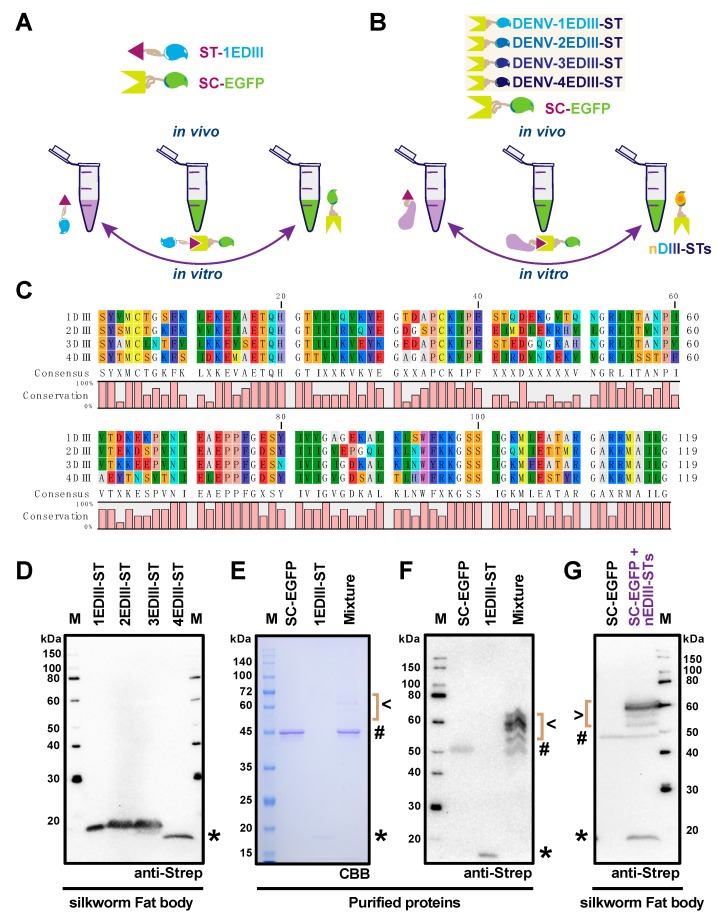
Protein ligation and labeling using silkworm-Spy-BEVS. The scheme of in vitro and in vivo protein ligations was shown in (**A**) (ST-1EDIII) and (**B**) (ST-nEDIIIs) using SC-EGFP. The amino acid alignment of dengue virus E protein domain III (EDIII) from four serotypes was shown in (**C**). The expression of each EDIII-ST in the fat body in baculovirus-infected silkworm larvae was verified by Western blot (**D**). The purified SC-EGFP and 1EDIII-ST were mixed in vitro and the ligated adduct was verified by either CBB-staining (**E**) or Western blot by anti-Strep (**F**). Super co-infection with five recombinant viruses containing SC-EGFP and four EDIII-STs (nEDIII-STs) was employed to explore the ability of in vivo ligation for multiple proteins of interest. The resulting products were identified by Western blot along with the SC-EGFP single infection control (**G**). # and * indicate SC-EGFP and nEDIIIs, respectively. Ligated products were marker by the brace and the completed adduct was marked by arrows.

To further explore the potential of SpyBEVS for protein labeling, as depicted in Figure 4A,B, the co-infection approach was utilized to test the flexibility and capacity in the presence of single or multiple rBmNPVs expressing ST-tagged POIs together with the SC-EGFP partner. In this study, we have selected to express and purify the domain III of E protein (EDIII) from four serotypes of the dengue virus, which have been reported as vital domains holding distinct immunogenicity [32]. The alignment of the amino acid sequence has been demonstrated in Figure 4C. All of these EDIIIs are C-terminal genetically fused with ST, termed 1EDIII-ST, 2EDIII-ST, 3EDIII-ST, and 4EDIII-ST, respectively. The soluble expression of each EDIII-ST in the fat body lysates in baculovirus-infected silkworm larvae has verified by Western blot (Figure 4D). As an example, the purified SC-EGFP and 1EDIII-ST proteins were mixed in vitro and the ligated adducts could be observed only after reaction for 1 h at room temperature, which was verified by either CBB-staining (Figure 4E) or Western blot by anti-Strep tag (Figure 4F). Remarkably, super co-infection with five recombinant viruses containing SC-EGFP and four EDIII-STs (nEDIII-STs) was subsequently employed to explore the ability of in vivo ligation for multiple POIs (Figure 4G). The resulting products were validated by Western blot along with the SC-EGFP single infection control, SC-EGFP and nEDIIIs, respectively, suggesting that sufficient, robust, and specific protein–protein conjugation could occur using the SpyBEVS platform.

## 3. Discussion

Recently, peptide-protein reactive pairs, such as ST/SC and SnoopTag/SnoopCatcher, have been considered as a versatile way for the bioconjugation of functional proteins as there are no catalytic enzymes or cofactors required [17,33,34]. Since the discovery of ST/SC chemistry, it has been validated by many researchers that in vivo and in vitro protein coupling could be achieved in several organisms including *E. coli*, yeast, and cultured mammalian cells [1,2,3]. This stable linkage of ST/SC ensures its applications in protein topology engineering and synthetic vaccine development. In this study, we have successfully established and evaluated the Silkworm-SpyBEVS, an in vivo protein conjugation platform using lepidopteran silkworms. As judged by the ligated adducts, we have obtained a proficient conjugation efficacy by mixing purified SC/ST protein pairs together in vitro, which is expected as other protein expression systems [17,23,30]. It was reported that the ST was reactive regardless of its terminal location, of N-internal or C-terminus [17]. Taken together with the results from Figure 3 and Figure 4, we have also proven that the location of ST is adjustable and largely depends on the property of POIs. In this study, we have observed that multiple conjugated products are generated either in vivo or in vitro ligation of SC-mCherry and ST-EGFP proteins. Although we could not figure out the exact reasons causing this phenomenon, it can be significantly improved when other POIs are applied, pointing out the importance of the initial design of bioconjugation partners.

It has been demonstrated that the ST/SC-based protein cyclization (SpyRing) of certain POIs (case-by-case), such as beta-lactamase and dihydrofolate reductase, confers higher thermal stability and activity [15,35,36]. Since there has been already some cases that recombinant proteins from silkworm-BEVS hold better stability and activity, further incorporation of SpyRing system might have a synergistic effect [37,38]. We are now challenging the silkworm-SpyRing-BEVS using some POIs in our laboratory. The further application of the SpyBEVS established in this study is also promising because the genetically coded pFastBac-Spy vectors (Figure 1B and Figure S1) are also compatible with other recombinant baculovirus-based protein expression systems, e.g., HiFive and Sf9 cells. Additionally, we have also developed a secreted SpyBEVS type (SecSpyBEVS, Figure S1) incorporating a signal peptide from silkworm 30 kDa lipoprotein [39], allowing the bioreaction selectively in the cultured medium or individual hemolymph. Further optimizations should be performed since no secreted SC-tagged POI could be detected in our preliminary experiments (data not shown).

It is significant to observe that the co-infection method brings the SC/ST proteins together sufficiently inside of the silkworm, either larvae or pupae. The well-established co-infection approach is a quite easy handling procedure in both cultured lepidopteran cell- and individual-based BEVS [4]. Recently, a SpyLigase-mediated ligation system developed by Fierer et al. (2014) significantly reduced the size of SC into two parts, SpyLigase, and K-Tag [40]. The co-infection technique could support this newly established ligation system using three rBmNPV viruses holding the corresponding ST/SpyLigase/K-Tag respectively. As demonstrated in Figure 4 of the current research, we have co-infected silkworm larvae with two or five different recombinant baculoviruses and obtained clear fluorescent EGFP-conjugated adducts. Distributable fusion tags (e.g., Flag, His8, Myc, and PA) could be further engineered to trace the yields of each conjugated adduct. This multiple protein labeling system in silkworm should be useful for protein imaging and tracing purposes in vivo. Furthermore, there have been several works describing the reported plug-and-display system for generating antigen-displaying virus-like particle (VLP) [22,23,34,41]. The SpyBEVS can produce either non-enveloped VLP or enveloped VLP to have multiple antigens assembled within silkworm and displayed on the VLP surfaces spontaneously. As described above, the silkworm-BEVS is considered as a large-scale protein production platform and thus has a high potential to be used for commercial purpose, which should accelerate vaccine development for many pathogenic diseases [5]. Collectively, our current study verified that the Spy-BEVS is working well for in vitro and in vivo protein bioconjugations, although further validation and improvement of this system are still required in the future.

## 4. Materials and Methods

### 4.1. Cultured Cells and Silkworms

The cultured silkworm Bm5 cells were cultured in Sf-900II medium (ThermoFisher Scientific K. K., Tokyo, Japan) supplemented with of 10% fetal bovine serum (Gibco, Tokyo, Japan) and 1% antibiotic-antimycotic (ThermoFisher Scientific K. K., Tokyo, Japan) at 27 °C. The fifth instar silkworm larvae were purchased from Ehime Sansyu (Ehime, Japan) and were reared with an artificial diet Silkmate S2 (Nosan, Yokohama, Japan) under controlled environmental conditions (25 °C, 65 ± 5% relative humidity).

### 4.2. Synthetic DNA for SpyTag/SpyCatcher Constructs

The insect codon-optimized DNA sequences of SpyTag/SpyCatcher002 [18] with tags were synthesized by Genewiz (Suzhou, China). The detailed information was provided as a Supplemental Figure S1. The synthesized DNA was then amplified by PCR (KOD-Plus-Neo DNA polymerase, Toyobo, Tokyo, Japan) using specific primers which were listed in Table 1. The amplified fragment was further sub-cloned into the pFastBac1 vector (ThermoFisher Scientific. K. K.). The resulting plasmids were termed pFastBac1-ST-N, pFastBac1-ST-C, and pFastBac1-SC, respectively. Subsequently, the PCR products of coding sequence of mCherry, Venus, enhanced green fluorescent protein (EGFP), and domains III of E protein (EDIII) from four serotype of dengue virus (1EDIII, 2EDIII, 3EDIII, and 4EDIII) were inserted into the above pFastBac-SpyTag/SpyCatcher plasmids (see Supplemental Figure S1 for detailed information).

### 4.3. Generation of Recombinant Baculoviruses

The transfer pFastBac plasmid for each ST/SC construct was transformed into *E. coli* BmDH10Bac cells to generate the recombinant BmNPV bacmid [42]. The corresponding bacmid was then transfected into either cultured Bm5 cells or silkworm fifth instar larvae using 1,2-dimyristyloxypropyl-3-dimethyl-hydroxy ethyl ammonium bromide and cholesterol (DMRIE-C) transfection reagents (1 μL/2 μg bacmid DNA, ThermoFisher Scientific K. K.) or chitosan/bacmid system to obtain recombinant baculoviruses according to the protocols established previously [43]. The stock of high titer of viruses (P3) was used for the infection of silkworm cells and larvae was achieved by the series infection method.

### 4.4. In Vitro and In Vivo Ligation ST/SC Constructions in Cultured Silkworm Cells and Larvae

The recombinant baculoviruses (P3) were directly employed to infect/co-infect cultured cells in 6-well plate or injected/co-injected into the silkworm larvae on the 3rd of fifth instar. The fluorescent signals from virus-infected silkworm larvae and pupae were detected by fluorescent microscopy or directly under ultraviolet light (UV). At four dpi, cultured cells or fat body tissues from 20 silkworm larvae were collected and lysed in a lysis buffer (100 mM Tris–HCl pH 8.4, 0.15 M NaCl, 1 mM EDTA 0.1% NP-40) with complete EDTA-free protease inhibitor tablet (1 tablet/100 mL; Roche, Paris, France). The clear supernatant was subjected to sodium dodecyl sulfate-polyacrylamide gel electrophoresis (SDS-PAGE) and Western blot, following by protein purification following our previous protocols. Briefly, ST/SC-fused proteins were separated on 12% SDS-PAGE gel and then transferred onto a polyvinylidene fluoride (PVDF) membrane using a semidry transfer cell (Bio-Rad, Hercules, CA, USA). The membrane was then blocked in a 5% (*w*/*v*) skimmed milk (Wako, Tokyo, Japan) containing TBST buffer (0.1% (*v*/*v*) Tween 20, Tris-buffered saline) for 1 h at room temperature. Subsequently, the membrane was incubated with anti-StrepTagII (1:5000, anti-mouse, QIAGEN, Hilden, Germany) or anti-DDDDK (1:5000, anti-mouse, MBL, Nagoya, Japan) primary antibody, followed by incubation with HRP-conjugated secondary antibody (1:10,000, GE Healthcare, Piscataway, NJ, USA). The final development was carried out using Immobilon western chemiluminescence HRP substrate (Merck Millipore, Darmstadt, Germany) and the images were gained by VersaDoc MP imaging systems (Bio-Rad).

### 4.5. Protein Purification and In Vitro Protein Ligation Assay

After verification of the expression level for each ST/SC-tagged protein, the fat body lysates from baculovirus-infected silkworm (~20) were further employed for protein purification procedures. The lysates were centrifuged at 8000× *g* for 30 min at 4 °C to remove the insoluble matter, followed by filtration with a 0.45-μm filter (Millipore). The clear supernatant was then diluted in a binding buffer (100 mM Tris–HCl pH 8.4, 0.15 M NaCl, 1 mM EDTA) and applied to a StrepTrap column (QIAGEN, Hilden, Germany). After being carefully washed, the target protein was eluted by a binding buffer containing 2.5 mM desthiobiotin (IBA Lifesciences, Germany). The purified proteins were concentrated by Amicon Ultra-15 (MWCO 30 kDa, Sigma, Millipore) and buffer-exchanged in PBS buffer, which was stored at −20 °C until use. The final protein concentration of purified protein samples was measured by BCA protein quantification kit (Bio-rad). The ST/SC-tagged proteins, for example, SpyCatcher-EGFP and NSpyTag-mCherry, were mixed and adjusted to a final 20 μL volume with PBS. The ligation reaction was performed at room temperature (~25 °C) for at least 1 h. The ligation efficiency was evaluated by SDS-PAGE or Western blot to investigate the formed adduct between ST and SC.

## Figures and Tables

**Table 1:** Primers used in this study.

Name	Sequence (5′-3′)
EGFP/Venus-Fw	gtgagcaagggcgaggagctg
EGFP/Venus-Rv-Stop	ccc*aagctt*ctacttgtacagctcgtccatg
EGFP/Venus-Rv-nStop	ccc*aagctt*cttgtacagctcgtccatgcc
mCherry-Fw	gtgagcaagggcgaggaggat
mCherry-Rv-Stop	ccc*aagctt*ctacttgtacagctcgtccatg
mCherry-Rv-nStop	ccc*aagctt*cttgtacagctcgtccatgcc
1DIII-Fw	agttatgttatgtgcaccgg
1DIII-Rv	gcccaaaatagccattcgcc
2DIII-Fw	tcatactctatgtgcacagg
2DIII-Rv	acctaaaatggccattctct
3DIII-Fw	tcttatgctatgtgtttgaa
3DIII-Rv	tcccaggatagccattcggc
4DIII-Fw	tcgtacactatgtgttcagg
4DIII-Rv	tcctagtattgccatacgct

Underlined are *HindIII* digestion site.

## Supplementary Materials

Supplementary materials can be found at https://www.mdpi.com/1422-0067/20/17/4228/s1. Supplementary Figure S1 Detailed information for SpyTag/SpyCatcher containing pFastBac Vectors developed in this study.

## Abbreviations

ST/SC	SpyTag/SpyCatcher
BEVS	Bacmid expression vector system
SpyBEVS	SpyTag/SpyCatcher-Bacmid expression vector system
FS	Flag-Strep-tag II
HS	His_8_-Strep-tag II
EDIII	Domain III of E dengue virus
POI	Protein of interest
DMRIE-C	1,2-dimyristyloxypropyl-3-dimethyl-hydroxy ethyl ammonium bromide and cholesterol
