# Supplementary material for: Development of SpyTag/SpyCatcher-Bacmid Expression Vector System (SpyBEVS) for Protein Bioconjugations Inside of Silkworms
Source: Int J Mol Sci. 2019 Aug 29;20(17):4228. doi: 10.3390/ijms20174228 (PMC6747175; doi:10.3390/ijms20174228)
Supplement: Supplementary file 1 [file ijms-20-04228-s001.pdf]

SuppFig. 1

pFastBac::L21>30k6G(±)–His8–Strep–Tag II–TEV–SpyCatcher002–*StuI*

aactcctaaaaaaccgccacc>atgagactgactttgttcgctttcgtgctggccgtgtgcgctctggcctcaaacgctca
ccaccaccaccatcaccatcacggaggaggttcagcatggtcacaccctcagttcgaaaagggaggaggatcagaga
atttatacttccaaggtggaggaggttcagtgactactctgtctggtctgtctggtgagcaaggtccttctggtgacatgaca
acagaagaggactcagccactcacatcaagttctcaaagcgcgacgaggacggtagagaactggccggtgctacaat
ggagctgcgcgactcttctggtaaaacaatctctacatggatctcagacggacatgtgaaggacttctatttataccccggt
aagtacactttcgtggagacagctgctcccgatggttacgaggtggccacagccatcacattcacagtgaacgagcaag
gtcaagttacagtgaacggtgaagccactaaaggagacgcccacactggatcatctggttctggtggatctggt*aggcct*

aactcctaaaaaaccgccacc>MRLTLFAFVLAVCALASNAHHHHHHHHGGGSAWSHPQFEKGG
GSENLYFQGGGGSVTTLSGLSGEQGPSGDMTTEEDSATHIKFSKRDEDGRELAGATMELR
DSSGKTISTWISDGHVKDFYLYPGKYTFVETAAPDGYEVATAITFTVNEQGQVTVNGEATKG
DAHTGSSGSGGSG*aggcct*

↑ EGFP; Venus……

pFastBac::L21>30k6G(±)–Flag–Strep–Tag II–TEV–SpyTag002–*StuI*

aactcctaaaaaaccgccacc>atgagactgactttgttcgctttcgtgctggccgtgtgcgctctggcctcaaacgctga
ctacaaggacgacgacgacaaaggtggtggttcagcatggtcacaccctcagttcgagaagggtggtggttcagagaat
ttatacttccaaggttcacagcccgttcctacaatcgtgatggtggacgcctacaagcgctacaagggttcatctggttctg
gtggttctggt*aggcct*

aactcctaaaaaaccgccacc>MRLTLFAFVLAVCALASNADYKDDDDKGGGSAWSHPQFEKGG
GSENLYFQGSQPVPTIVMVDAYKRYKGSSGSGGSG*aggcct*

↑ mCherry

pFastBac::L21>30k6G(±)–*StuI*–SpyTag002–TEV–Strep–Tag II–Flag

aactcctaaaaaaccgccacc>atgagactgacactgttcgctttcgtgctggctgtgtgcgctctggcttcaaacgct*ag*
*gcct*tcacagcccggttcatctggttctggtggttctggtgtgcctacaatcgtgatggtggacgcttacaagcgctacaaa
ggtggtggttcagagaatttatacttccaaggtggtggtggttcagcatggtcacaccctcagttcgagaagggtggtggtt
cagactacaaggacgacgacgacaaataa

↓ mCherry; Venus; EDIIIs

aactcctaaaaaaccgccacc>MRLTLFAFVLAVCALASNA*aggcct*SQPGSSGSGGSGVPTIVMVD
AYKRYKGGGSENLYFQGGGGSAWSHPQFEKGGGSDYKDDDDK*

→ *StuI* cloning site, combining with *e.g. HindIII* site from pFastBac

→ Single *StuI* cloning site, dephosphorylation to prevent self-ligation
Phosphorylation is required for inserts

Xu et al., 2019
